# Concomitant Usage of H1‐Antihistamines and Immune Checkpoint Inhibitors on Cancer Patient Survival

**DOI:** 10.1002/cam4.70583

**Published:** 2025-01-10

**Authors:** Yin Leung, Terry Cheuk‐Fung Yip, Grace Lai‐Hung Wong, Vincent Wai‐Sun Wong, Vicki Wing‐Ki Hui, Tony Shu‐Kam Mok, Henry Lik‐Yuen Chan, Stephen Lam Chan, Rashid Nok‐Shun Lui

**Affiliations:** ^1^ Medical Data Analytics Centre The Chinese University of Hong Kong Hong Kong SAR China; ^2^ Department of Medicine and Therapeutics The Chinese University of Hong Kong Hong Kong SAR China; ^3^ Institute of Digestive Disease The Chinese University of Hong Kong Hong Kong SAR China; ^4^ State Key Laboratory of Translational Oncology, Department of Clinical Oncology The Chinese University of Hong Kong Hong Kong SAR China

## Abstract

**Purpose:**

Recent research (Li et al. 2021) suggests an upregulated expression and activation of H1 receptors on macrophages in the tumor microenvironment, and concomitant H1‐antihistamine use is associated with improved overall survival in patients with lung and skin cancers receiving immunotherapy. Therefore, we retrospectively evaluated the impacts of H1‐antihistamine use in cancer patients during immunotherapy.

**Methods:**

All patients who had received at least one dose of immune checkpoint inhibitors (ICIs) from July 1, 2014 to October 31, 2019 were identified from Hong Kong's territory‐wide database, with this date defined as the baseline. A 1‐month landmark analysis was conducted with follow–for up to 6 months, including an exposure period of 1 month before and after the baseline date. Patients were grouped according to the types of primary cancer and the percentages of daily H1‐antihistamine usage within the exposure period. The primary outcome was overall survival.

**Results:**

A total of 1740 (65.1% male, mean age 61.9 years) were included in the landmark analysis, of which 529 (30.4%) and 307 (17.6%) had primary lung and liver malignancies. The multivariable Cox regression model estimated statistically significant improvement in overall survival of intermediate use in patients with primary lung malignancies (adjusted hazard ratio [aHR] 0.223, 95% confidence interval [CI] 0.052–0.958, *p* = 0.044), but not with primary liver maligancies. Similar frequency‐dependent effects were identified in Kaplan–Meier analysis.

**Conclusion:**

The benefits of adjunctive use of H1‐antihistamines may be generation‐ and tumor‐dependent. Further clinical and mechanistic studies are required to confirm the findings.

## 1 Introduction

Immune checkpoint inhibitors (ICIs) are a type of immunotherapy that counteracts T cell inhibition mediated by the PD‐L1/PD‐L2‐PD‐1 and CD80/CD86‐CTLA‐4 co‐inhibitory signaling pathways. Since their first introduction and approval in the last decade, there have been rapid developments in the types of monoclonal antibodies and an increasing expansion of indications across many malignancy types [1, 2, 3]. Recently, our understanding of biomarkers has led to the incorporation of tumor‐agnostic indications for ICIs, such as for tumors that exhibit microsatellite instability‐high/deficient mismatch repair or tumor mutational burden‐high, regardless of the anatomical origin of the cancer.

Recent translational research [4] suggests that upregulation of both histamine and the histamine receptor H1 (HRH1) in the immunosuppressive tumor microenvironment (TME) occurs with the polarization of tumor‐associated macrophages (TAMs) toward the M1‐like phenotype by HRH1 activation. HRH1 blockade reverses this induced T cell dysfunction by downregulating the immune checkpoint VISTA on TAMs. Retrospective evaluation of the concomitant use of H1‐antihistamines during ICI treatment in a study from Texas, United States [4] found statistically significant improvements in overall survival for lung and skin malignancies, and similar trends were found in breast and colon malignancies despite the absence of statistical significance.

Current indications for H1‐antihistamine use [5] could be widely categorized into allergic and nonallergic disorders. For first‐generation H1‐antihistamines, these primarily include nausea, vomiting, and vertigo secondary to motion sickness, whereas second‐generation antihistamines are now considered the medication of choice for most allergic disorders, such as allergic rhinitis, urticaria, and allergic conjunctivitis, because they do not readily cross the blood–brain barrier unlike the first‐generation drugs, making them exhibit fewer off‐target side effects, such as drowsiness. Third‐generation antihistamines are metabolites or enantiomers of the previous generations and have been proven to be safer and have a faster onset of action than older agents.

This retrospective territory‐wide cohort study aims to explore whether concomitant H1‐antihistamine use in patients with malignancies treated with ICIs has an impact on patient survival using real‐world data.

## 2 Methods

### 2.1 Data Source

This study was conducted using data retrieved from the Clinical Data Analysis and Reporting System (CDARS) managed by Hong Kong's Hospital Authority (HA). The registry captures clinical data of all patients attending the 43 hospitals and 122 outpatient clinics under HA, and the records represent approximately 80% of all inpatient and outpatient visits of the 7.4‐million local population [6, 7]. This system enables accurate and comprehensive retrieval of clinical data that supports retrospective clinical and management decisions for analysis and reporting.

Subjects are de‐identified and linked with unique reference keys to protect patient privacy. Clinical data from CDARS have previously been used in epidemiological research projects [8, 9]. Patient diagnoses are coded by the International Classification of Diseases, Ninth Revision, Clinical Modification (ICD‐9‐CM), and the accuracy of code usage has been shown to approach 99% in a prior study [10].

### 2.2 Study Population

All consecutive patients who received at least one dose of ICIs from July 1, 2014 to December 31, 2019 were first identified. Patients without a date of birth or those below the age of 18 years were then excluded. The primary outcome was overall survival. The remaining patients in the cohort were followed from the date of their first prescription of ICI to the date of death from any cause, censored at the date of their last prescription of H1‐antihistamines before June 1, 2022.

Their demographic characteristics (sex and date of birth), neoplasm type, relevant comorbidities, and clinical laboratory results were collected. Baseline liver and renal function biochemistries and hematological and virological (e.g., hepatitis B surface antigen [HBsAg], antibody to hepatitis C virus [anti‐HCV]) parameters were determined by the laboratory results obtained immediately prior to the first dose of ICI. Prescription and dispensing records of H1‐antihistamines from August 1, 2001 to June 1, 2022 were also collected.

The study protocol was approved by the Joint Chinese University of Hong Kong—New Territories East Cluster Clinical Research Ethics Committee (CREC Reference Number: 2018.647). Informed consent was waived due to the retrospective nature of the study.

### 2.3 Use and Indications of ICIS

The ICIs that patients were treated with targeted three types of molecules: (1) anti‐programmed death‐1 (PD‐1) antibodies: pembrolizumab and nivolumab; (2) anti‐programmed death‐ligand 1 (PD‐L1) antibodies: atezolizumab, avelumab, and durvalumab; and (3) anti‐cytotoxic T lymphocyte‐associated antigen 4 (CTLA‐4) antibodies: ipilimumab. Patients could be prescribed monotherapy, combination therapy, or sequential therapy ICIs.

Categorization of malignancy types was based on the primary site or tissue of the malignant neoplasms only (see Table S1); therefore, each patient would be coded with only one type of primary malignancy.

### 2.4 H1‐Antihistamine Exposure

Baseline was defined as the date of the first prescription of ICI. A 1‐month landmark analysis followed up to 6 months was performed to minimize immortal time bias and reverse causation. The landmark time and the time horizon at which patients were censored were selected pragmatically by considering the pharmacokinetic profile of H1‐antihistamines and the indications for ICI use.

The time to maximum plasma concentration (*T*max^c^) of oral H1‐antihistamines of any generation is within 3 h, and the terminal plasma half‐life (*t*_1/2_) peaks at 28 h for the first generation but peaks at around 12 h for the second‐ and third‐generation agents [5]. The choices of both time points were further validated by the median of the lengths of the time interval between the baseline and the date of occurrence of the event of interest (all‐cause mortality) or censoring event (last prescription of H1‐antihistamines).

The exposure period to H1‐antihistamines was determined as 1 month before and after the baseline. There are currently no approved malignancy‐specific indications for the use of H1‐antihistamines. Therefore, the concept of medication possession ratio (MPR) [11], a commonly adopted approach to estimate medication adherence and persistence by calculating the proportion of days' supply available within the observation period, was referenced to determine the stratification of H1‐antihistamine users.

Patients were categorized by the quartiles of percentages of days' supply during the exposure period into (1) minimal users (0%–25%), (2) infrequent users (25%–50%), (3) intermediate users (50%–75%), and (4) frequent users (75%–100%). The categorization was determined using the formula that divides the total number of discontinuous days' supply of H1‐antihistamines by the exposure period, defined as the total number of days accumulated from 1 month before to 1 month after the baseline, defined as the date of the first prescription of ICI mentioned above. Patients who were never exposed to H1‐antihistamines or only took H1‐antihistamines before and after the exposure period were included as minimal users. A similar stratification criterion was adopted in a prior study on other underexplored clinical indications of H1‐antihistamines [12].

Patients were also classified according to the generation of H1‐antihistamines prescribed: (1) first‐ (chlorpheniramine, diphenhydramine, hydroxyzine, and promethazine) and/or second‐ (cetirizine and loratadine) generation users, and (2) third‐ (fexofenadine) with or without first‐ and/or second‐generation H1‐antihistamine usage. Such categorization is due to the common clinical practice to switch to an alternative generation of H1‐antihistamines for patients who do not respond, exhibit antihistamine resistance, or simply to restore efficacy. Although patients may have taken multiple generations of H1‐antihistamines during the exposure period, the groupings encompassed all possible combinations, and each patient would only be assigned to one grouping.

### 2.5 Statistical Analysis

Data were analyzed using R software (4.2.1; R Foundation for Statistical Computing, Vienna, Austria). All statistical tests were two‐sided with statistical significance taken as *p* < 0.05.

For the interpretation of baseline characteristics, continuous variables were expressed in mean ± standard deviation or median (interquartile range [IQR]) as appropriate, whereas categorical variables were expressed as number (percentage). Quantitative and qualitative differences between subgroups were analyzed by one‐way ANOVA for continuous parameters or the Kruskal–Wallis rank‐sum test for categorical parameters, where appropriate.

The Cox proportional hazards regression analyses were run to estimate the unadjusted hazard ratios and adjusted hazard ratios (aHRs) (adjusted for baseline characteristics and prescription and dispensing records of H1‐antihistamines) and associated 95% confidence intervals (CIs). The predefined covariates at baseline included in the analyses were age, sex, neutrophil‐to‐lymphocyte ratio (NLR), platelet‐to‐lymphocyte ratio (PLR), days' supply and generation type of H1‐antihistamines, and the types of ICIs. Patients with missing values of NLR and PLR were excluded from the analyses. Backward elimination was also implemented to remove insignificant covariates in the multivariable analyses. Subgroup analyses on patients with (1) primary lung malignancies, (2) primary liver malignancies, and (3) other primary malignancies were performed.

A sensitivity analysis of a 2‐month H1‐antihistamine exposure period before and after the baseline, followed up to 6 months, was performed to eliminate the possibility of data‐driven results and to recognize that the probability estimate of death from any cause is conditional on the definitions of the study population and the H1‐antihistamine exposure period at the landmark time. The Kaplan–Meier method was used to estimate the cumulative probability of death from any cause. The log‐rank test was also used to compare the probabilities at every time point of the time horizon and the overall 6‐month survival curves between the users grouped by the quartiles of days' supply of H1‐antihistamines.

## 3 Results

### 3.1 Baseline Characteristics

**FIGURE 1:**
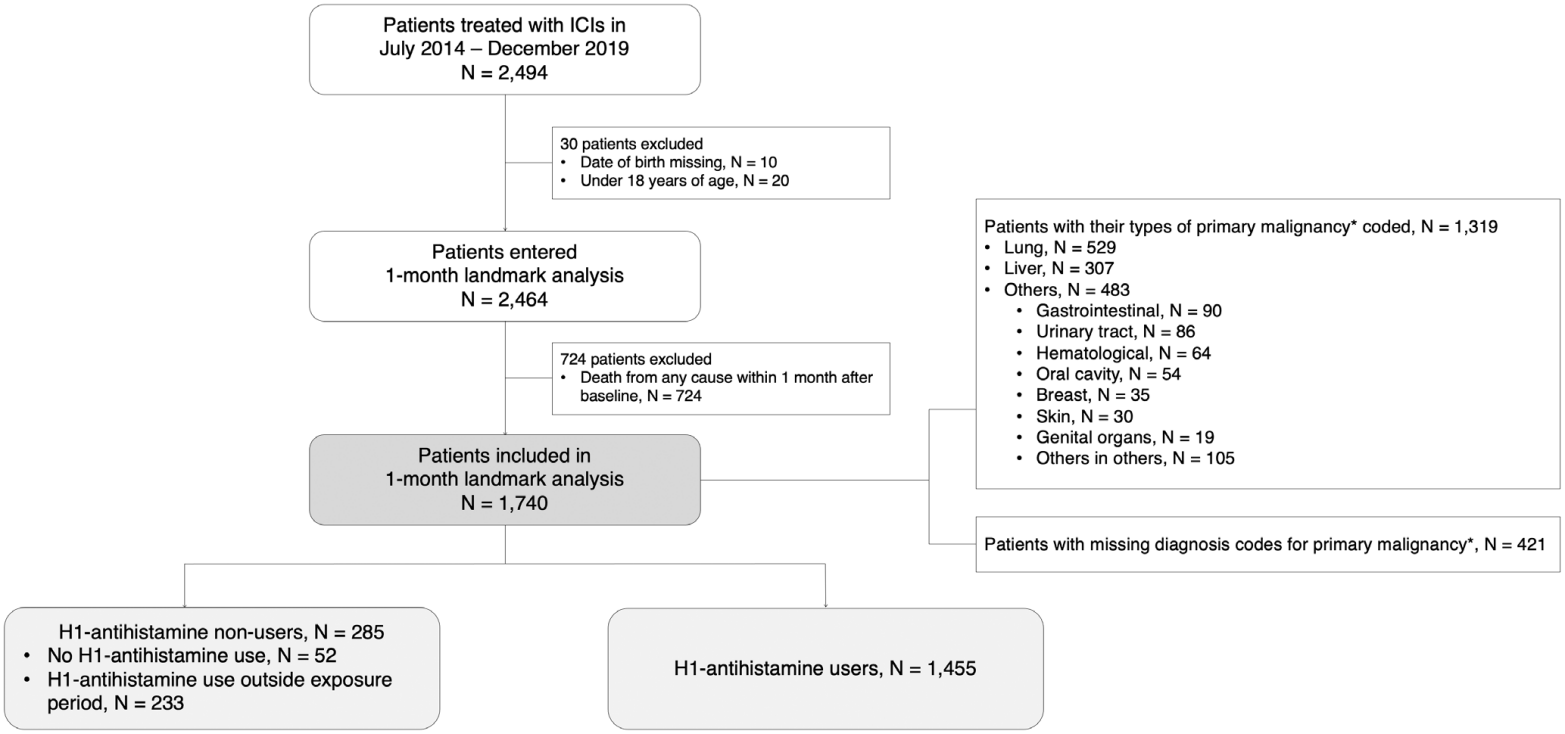
Study flowchart detailing patient identification and inclusion criteria of the landmark analysis. *Categorization of malignancy types was based on the primary site or tissue of the malignant neoplasms only.

Among the 2494 patients identified who received ICIs, 30 were excluded due to missing date of birth or age under 18 years. After excluding subjects who died within 1 month from baseline, 1740 patients were included in the landmark analysis. There were 1455 (83.6%) H1‐antihistamine users in the cohort, and the remaining 285 (16.4%) either had no recorded H1‐antihistamine use or took H1‐antihistamines outside the exposure period (see Figure 1).

Of all the patients who fulfilled the inclusion criteria of the landmark analysis (see Figure 1), 1319 (75.8%) patients had their types of primary malignancies coded. Primary lung (*N* = 529; 30.4%) and primary liver (*N* = 307; 17.6%) malignancies were the most common cancers in our cohort. Primary malignancies of the gastrointestinal, urinary tract, hematological, oral cavity, breast, skin, and genital organs represented the majority of the remaining cancers (*N* = 378; 21.7%). Individual cohort sizes for these types of malignancies were too small to support further subgroup analyses. There were 421 (24.2%) patients with a missing diagnosis code for their type of primary malignancy.

According to the days' supply of H1‐antihistamines within the exposure period, 1377 (79.1%) were minimal users, 168 (9.7%) were infrequent users, 87 (5.0%) were intermediate users, and 108 (6.2%) were frequent users (see Table 1). The patients were followed for a median of 7.0 months (IQR 3.1–15.4 months), with shorter follow‐ups in intermediate (4.6 months) and frequent (5.7 months) users. These numbers justified the choice of the time horizon in the landmark analysis.

**TABLE 1:** Baseline characteristics of patients included in the 1‐month landmark analysis, grouped according to the quartiles of percentages of days' supply of H1‐antihistamines during the exposure period.

	All	H1‐antihistamine users				*p*
		Minimal	Infrequent	Intermediate	Frequent	
	(*N* = 1740)	(*N* = 1377)	(*N* = 168)	(*N* = 87)	(*N* = 108)	
Duration of follow‐up (months)^a^	7.0 (3.1–15.4)	7.1 (3.1–15.5)	8.0 (3.7–16.7)	4.6 (2.2–11.8)	5.7 (2.8–12.2)	**0.035**
Demographic characteristics						
Male gender, *n* (%)	1133 (65.1)	899 (65.3)	108 (64.3)	53 (60.9)	73 (67.6)	0.793
Age (years)	61.9 ± 12.5	61.7 ± 12.6	61.7 ± 12.7	61.6 ± 12.5	64.8 ± 11.0	0.099
Clinical characteristics						
Hemoglobin (g/dL)	11.5 ± 2.0	11.5 ± 2.0	11.7 ± 2.0	11.3 ± 1.9	11.4 ± 1.7	0.432
White cell count (×10^9^/L)	7.5 ± 4.8	7.4 ± 4.6	7.2 ± 3.5	8.6 ± 8.7	8.1 ± 4.5	0.104
Neutrophil (×10^9^/L)	5.3 ± 3.7	5.3 ± 3.8	5.3 ± 3.2	5.2 ± 2.7	5.5 ± 3.4	0.953
Lymphocyte (×10^9^/L)	1.3 ± 1.2	1.3 ± 1.3	1.3 ± 1.3	1.1 ± 0.5	1.3 ± 0.7	0.713
Platelet (×10^9^/L)	270.6 ± 133.6	271.1 ± 133.4	249.8 ± 113.8	281.8 ± 153.3	287.8 ± 145.8	0.089
Neutrophil‐to‐lymphocyte ratio	5.4 ± 5.7	5.3 ± 5.5	5.6 ± 4.8	6.7 ± 10.3	5.4 ± 5.2	0.317
Missing (%)	25.7	26.1	24.4	32.2	17.6	
Platelet‐to‐lymphocyte ratio	267.1 ± 202.1	265.3 ± 191.9	253.0 ± 150.0	340.0 ± 400.3	260.0 ± 179.0	**0.037**
Missing (%)	25.7	26.1	24.4	32.2	17.6	
International normalized ratio	1.1 ± 0.2	1.1 ± 0.2	1.1 ± 0.1	1.1 ± 0.1	1.1 ± 0.2	0.863
C‐reactive protein (mg/dL)	46.3 ± 52.5	46.1 ± 52.9	45.9 ± 39.7	45.2 ± 48.4	49.4 ± 64.3	0.989
Albumin (g/L)	35.8 ± 6.3	35.9 ± 6.3	36.2 ± 6.3	35.1 ± 6.9	35.1 ± 6.8	0.344
C‐reactive‐protein‐to‐albumin ratio	1.5 ± 2.0	1.5 ± 2.0	1.5 ± 1.5	1.3 ± 1.5	1.8 ± 3.3	0.847
Total bilirubin (μmol/L)	12.4 ± 25.2	12.2 ± 25.7	10.7 ± 10.5	21.7 ± 41.7	9.8 ± 10.7	**0.003**
Alanine aminotransferase (U/L)	21.2 (14.0–36.0)	21.0 (14.0–36.0)	23.0 (16.0–36.2)	22.0 (16.0–57.0)	21.0 (14.0–33.0)	0.085
Aspartate aminotransferase (U/L)	37.0 (24.9–99.0)	37.0 (24.0–88.0)	60.7 (30.5–154.0)	37.0 (25.5–84.0)	30.0 (25.8–71.8)	0.976
Missing (%)	68.6	69.1	67.3	69.0	63.9	
Creatinine (μmol/L)	78.4 ± 39.2	77.7 ± 33.6	76.2 ± 41.2	81.8 ± 28.3	88.5 ± 83.9	**0.031**
Alpha‐fetoprotein (μg/L)	3.1 (2.0–34.1)	3.2 (2.0–37.6)	3.1 (1.9–126.9)	3.0 (1.8–16.5)	2.5 (2.0–4.3)	0.731
Missing (%)	52.5	53.4	49.4	46.0	50.0	
Positive HBsAg, *n* (%)^b^	62 (15.8)	43 (14.1)	9 (24.3)	6 (30.0)	4 (13.3)	0.117
Missing (%)	77.4	77.8	78.0	77.0	72.2	
Positive anti‐HCV, *n* (%)^b^	4 (1.5)	4 (1.9)	0 (0.0)	0 (0.0)	0 (0.0)	0.776
Missing (%)	84.7	84.8	84.5	85.1	83.3	
ICI type, *n* (%)^c^						
PD‐1						
Pembrolizumab	890 (51.1)	692 (50.3)	94 (56.0)	49 (56.3)	55 (50.9)	0.491
Nivolumab	597 (34.3)	475 (34.5)	63 (37.5)	26 (29.9)	33 (30.6)	0.515
PD‐L1						
Atezolizumab	251 (14.4)	202 (14.7)	18 (10.7)	12 (13.8)	19 (17.6)	0.391
Avelumab	2 (0.1)	1 (0.1)	0 (0.0)	1 (1.1)	0 (0.0)	**0.036**
Durvalumab	7 (0.4)	5 (0.4)	1 (0.6)	1 (1.1)	0 (0.0)	0.609
CTLA‐4						
Ipilimumab	157 (9.0)	107 (7.8)	32 (19.0)	10 (11.5)	8 (7.4)	**< 0.001**
ICI regimen, *n* (%)						
PD‐1 alone	1236 (71.0)	986 (71.6)	112 (66.7)	60 (69.0)	78 (72.2)	0.336
PD‐L1 alone	219 (12.6)	177 (12.9)	15 (8.9)	11 (12.6)	16 (14.8)	0.428
CTLA‐4 alone	2 (0.1)	2 (0.1)	9 (0.0)	0 (0.0)	0 (0.0)	0.911
PD‐1/PD‐L1 ± CTLA‐4	1455 (83.6)	1163 (84.5)	127 (75.6)	71 (81.6)	94 (87.0)	**< 0.001**

*Note:* Duration of follow‐up, alanine aminotransferase, aspartate aminotransferase, and alpha‐fetoprotein were expressed as median (interquartile range), whereas other continuous variables were expressed as mean ± standard deviation. *p* < 0.05 were underlined and made bold.

Abbreviations: anti‐HCV, antibody to hepatitis C virus; HBsAg, hepatitis B surface antigen; ICI, immune checkpoint inhibitor.

^a^ Duration of follow‐up was the duration from the date of the first prescription of ICI to the date of death from any cause, censored at the date of their last prescription of H1‐antihistamines before June 1, 2022.

^b^ Percentages were based on non‐missing data only.

^c^ Patients may have combined or switched ICIs during the follow‐up period.

**FIGURE 2:**
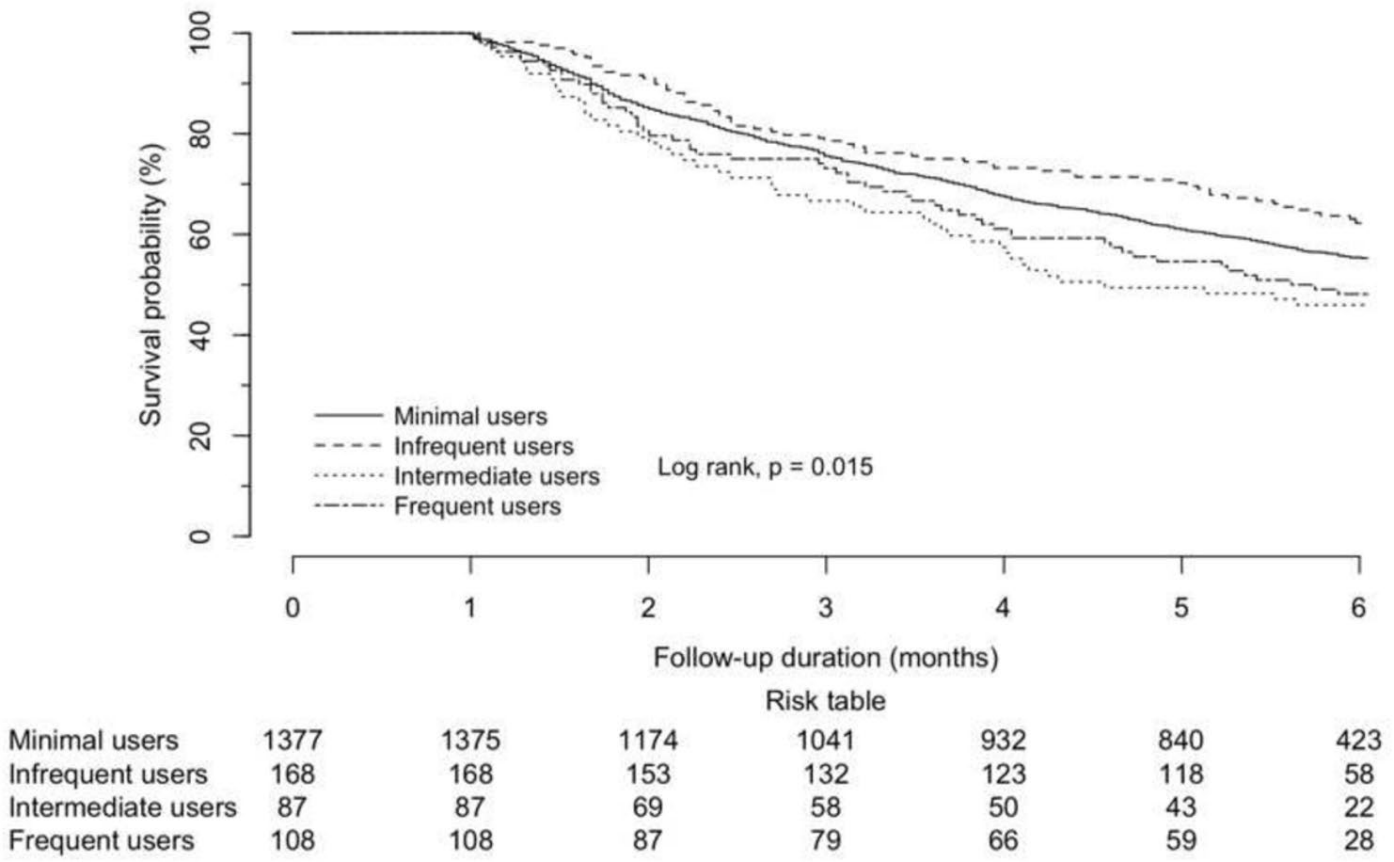
Kaplan–Meier survival curve of the 1‐month landmark analysis in all patients according to the quartiles of days' supply of H1‐antihistamines.

**FIGURE 3:**
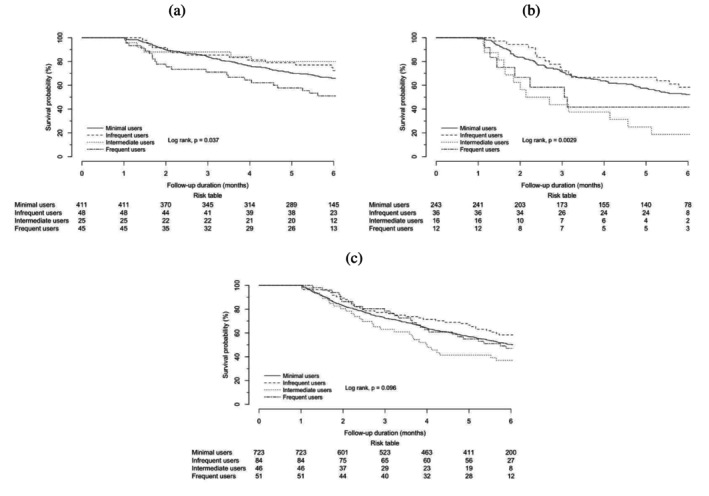
Kaplan–Meier survival curves of the 1‐month landmark analysis in patient subgroups: (a) patients with lung malignancies, (b) patients with liver malignancies, and (c) patients with missing diagnosis codes or other malignancies, according to the quartiles of days' supply of H1‐antihistamines.

Most patients were male (65.1%) with a mean age of 61.9 years (see Table 1). There were 25.7% of missing values in both NLR and PLR in all patients, and the percentage ranged from 17.6% to 32.2% across the H1‐antihistamine user groups categorized according to frequency. For hematological parameters, all except NLR, PLR, C‐reactive protein (CRP), and CRP‐to‐albumin (CRP/ALB) ratio fell within reference intervals. These are research‐identified inflammation‐based prognostic markers for malignancies, especially for lung and liver types, and the high values across all groups were consistent with the sustained inflammatory profile in the TME and subsequently poor prognosis indicative of ICI use [13, 14].

According to the malignancy type, 529 (30.4%) patients had primary lung malignancies, 307 (17.6%) patients had primary liver malignancies, and 904 (52.0%) patients had missing diagnosis codes or other types of primary malignancies (see Table 2). These findings were consistent with the distributions of ICI treatments received (see Table 1), which showed that most patients (around 70%) took anti‐PD‐1 antibodies across all groups of users.

**TABLE 2:** Types of H1‐antihistamine use in the cohort of the landmark analysis.

	All (*N* = 1740)	Type of malignancy of patients^a^		
		Lung (*N* = 529)	Liver (*N* = 307)	Missing or others (*N* = 904)
H1‐antihistamine use^c^	1455 (83.6)	436 (82.4)	259 (84.4)	760 (84.1)
First and/or second generation	1371 (78.8)	422 (79.8)	242 (78.8)	707 (78.2)
First generation				
Chlorpheniramine^b^	1390 (79.9)	419 (79.2)	246 (80.1)	725 (80.2)
Hydroxyzine	230 (13.2)	55 (10.4)	41 (13.4)	134 (14.8)
Promethazine	226 (13.0)	79 (14.9)	42 (13.7)	105 (11.6)
Diphenhydramine	161 (9.3)	51 (9.6)	15 (4.9)	95 (10.5)
Second generation				
Loratadine	386 (22.2)	121 (22.9)	59 (19.2)	206 (22.8)
Cetirizine	153 (8.8)	48 (9.1)	24 (7.8)	81 (9.0)
Third ± first and/or second generation	84 (4.8)	14 (2.6)	17 (5.5)	53 (5.9)
Third generation				
Fexofenadine	84 (4.8)	14 (2.6)	17 (5.5)	53 (5.9)

^a^ Categorization of malignancy types was based on the primary site or tissue of the malignant neoplasms only.

^b^ Chlorpheniramine included chlorpheniramine and dexchlorpheniramine.

^c^ Patients may switch to a different generation of H1‐antihistamines more than once during the exposure period.

As for the distribution of H1‐antihistamine use within the cohort (see Table 2), most patients (at least 82% across all subgroups of patients) were H1‐antihistamine users. The majority were prescribed first‐ and/or second‐generation H1‐antihistamines, with chlorpheniramine being the most commonly used drug. Approximately 5% received third‐generation H1‐antihistamines, with or without first‐ and/or second‐generation H1‐antihistamines. The pattern was consistent across the subgroups of patients with different primary malignancies.

**TABLE 3:** Univariate and multivariable analyses of factors associated with death from any cause within 6 months after receiving the first dose of ICI, with an exposure period to H1‐antihistamines 1 month before and after the baseline in all patients.

	Univariate analysis		Multivariate analysis	
	HR (95% CI)	*p*	aHR (95% CI)	*p*
All patients (*N* = 1740)				
Age (years)	0.990 (0.989–1.000)	**0.043**	0.993 (0.986–1.000)	0.056
Male gender, *n*	1.030 (0.889–1.193)	0.698		
Neutrophil‐to‐lymphocyte ratio	1.030 (1.027–1.043)	**< 0.001**	1.043 (1.031–1.056)	**< 0.001**
Platelet‐to‐lymphocyte ratio	1.000 (1.000–1.001)	**< 0.001**	1.000 (0.999–1.000)	0.058
By H1‐antihistamine days' supply				
Minimal user	Referent			
Infrequent user	0.789 (0.609–1.022)	0.073	0.866 (0.637–1.178)	0.360
Intermediate user	1.365 (1.015–1.837)	**0.040**	1.158 (0.774–1.732)	0.475
Frequent user	1.234 (0.938–1.622)	0.133	1.341 (0.966–1.861)	0.079
By H1‐antihistamine generation type^a^				
Nonuser	Referent			
First and/or second generation	1.054 (0.871–1.277)	0.587	0.989 (0.780–1.253)	0.927
Third ± first and/or second generation	0.688 (0.457–1.036)	0.074	0.584 (0.339–1.008)	0.054
By ICI type^b^				
PD‐1 only	Referent			
PD‐L1 only	1.047 (0.849–1.290)	0.669	1.153 (0.916–1.451)	0.226
CTLA‐4 ± PD‐1/PD‐L1	0.579 (0.441–0.760)	**< 0.001**	0.771 (0.563–1.056)	0.106

*Note:* *p* < 0.05 were underlined and made bold.

Abbreviations: aHR, adjusted hazard ratio; CI, confidence interval; HR, hazard ratio.

^a^ Patients may take multiple types of H1‐antihistamines during the exposure period.

^b^ Patients could be prescribed monotherapy, a combination, or a switch of ICIs, as indicated by their ICD‐9‐CM–coded malignancy types.

### 3.2 Impacts of Concomitant H1‐Antihistamine Use on Survival Outcomes

For patients with primary lung malignancies (see Table 3 and 4), intermediate use was observed to be an independent protective factor against mortality (aHR 0.223, 95% CI 0.052–0.958, *p =* 0.044), but frequent use was always an independent risk factor for mortality of statistical significance (aHR 1.723, 95% CI 1.024–2.901, *p* = 0.040) (Figures 2 and 3).

**TABLE 4:** Univariate and multivariable analyses of factors associated with death from any cause within 6 months after receiving the first dose of ICI, with an exposure period to H1‐antihistamines 1 month before and after the baseline in patient subgroups: (a) patients with primary lung malignancies, (b) patients with primary liver malignancies, and (c) patients with missing diagnosis codes or other primary malignancies.

	Univariate analysis		Multivariate analysis	
	HR (95% CI)	*p*	aHR (95% CI)	*p*
Patients with primary lung malignancies (*N* = 529)				
Age (years)	1.000 (0.991–1.018)	0.558		
Male gender, *n*	0.700 (0.517–0.937)	**0.017**		
Neutrophil‐to‐lymphocyte ratio	1.030 (1.014–1.043)	**< 0.001**	1.032 (1.014–1.049)	**< 0.001**
Platelet‐to‐lymphocyte ratio	1.000 (1.000–1.001)	**0.007**		
By H1‐antihistamine days' supply				
Minimal user	Referent			
Infrequent user	0.761 (0.431–1.344)	0.347	0.771 (0.401–1.483)	0.436
Intermediate user	0.565 (0.231–1.378)	0.209	0.223 (0.052–0.958)	**0.044**
Frequent user	1.670 (1.065–2.618)	**0.025**	1.723 (1.024–2.901)	**0.040**
By H1‐antihistamine generation type^a^				
Nonuser	Referent			
First and/or second generation	1.075 (0.730–1.584)	0.714	1.040 (0.662–1.634)	0.865
Third ± first and/or second generation	0.375 (0.090–1.566)	0.178	0.351 (0.047–2.639)	0.309
By ICI type^b^				
PD‐1 only	Referent			
PD‐L1 only	1.600 (1.168–2.193)	**0.003**	1.608 (1.128–2.291)	**0.008**
CTLA‐4 ± PD‐1/PD‐L1	0.358 (0.114–1.127)	0.079	0.290 (0.071–1.189)	0.086
Patients with primary liver malignancies (*N* = 307)				
Age (years)	0.990 (0.978–1.003)	0.121	0.981 (0.968–0.995)	**0.009**
Male gender, *n*	1.090 (0.718–1.652)	0.687		
Neutrophil‐to‐lymphocyte ratio	1.130 (1.072–1.196)	**< 0.001**	1.137 (1.074–1.203)	**< 0.001**
Platelet‐to‐lymphocyte ratio	1.000 (1.001–1.003)	**< 0.001**		
By H1‐antihistamine days' supply				
Minimal user	Referent			
Infrequent user	0.810 (0.473–1.387)	0.442	0.979 (0.541–1.773)	0.945
Intermediate user	2.609 (1.468–4.635)	**0.001**	2.585 (1.227–5.446)	**0.013**
Frequent user	1.576 (0.735–3.381)	0.242	1.557 (0.622–3.903)	0.345
By H1‐antihistamine generation type^a^				
Nonuser	Referent			
First and/or second generation	0.980 (0.633–1.518)	0.928	1.042 (0.607–1.787)	0.882
Third ± first and/or second generation	0.478 (0.182–1.253)	0.133	0.520 (0.171–1.582)	0.249
By ICI type^b^				
PD‐1 only	Referent			
PD‐L1 only	1.255 (0.398–3.953)	0.699	1.935 (0.601–6.232)	0.269
CTLA‐4 ± PD‐1/PD‐L1	0.658 (0.445–0.973)	0.036	0.844 (0.552–1.293)	0.436
Patients with missing diagnosis codes or other primary malignancies (*N* = 904)				
Age (years)	1.000 (0.989–1.003)	0.222	0.998 (0.989–1.008)	0.676
Male gender, *n*	1.220 (1.012–1.481)	**0.037**	1.101 (0.853–1.422)	0.459
Neutrophil‐to‐lymphocyte ratio	1.040 (1.033–1.054)	**< 0.001**	1.040 (1.027–1.054)	**< 0.001**
Platelet‐to‐lymphocyte ratio	1.000 (1.001–1.002)	**< 0.001**	1.000 (1.000–1.001)	0.533
By H1‐antihistamine days' supply				
Minimal user	Referent			
Infrequent user	0.778 (0.550–1.100)	0.155	0.835 (0.540–1.291)	0.417
Intermediate user	1.452 (0.995–2.121)	0.053	1.574 (0.962–2.574)	0.071
Frequent user	1.036 (0.701–1.532)	0.859	1.202 (0.743–1.943)	0.454
By H1‐antihistamine generation type^a^				
Nonuser	Referent			
First and/or second generation	1.054 (0.817–1.360)	0.686	0.952 (0.683–1.328)	0.773
Third ± first and/or second generation	0.746 (0.458–1.213)	0.238	0.686 (0.345–1.367)	0.284
By ICI type^b^				
PD‐1 only	Referent			
PD‐L1 only	0.984 (0.712–1.359)	0.921	1.112 (0.784–1.577)	0.553
CTLA‐4 ± PD‐1/PD‐L1	0.408 (0.263–0.634)	**< 0.001**	0.527 (0.294–0.947)	**0.032**

*Note: p* < 0.05 were underlined and made bold.

Abbreviations: aHR, adjusted hazard ratio; CI, confidence interval; HR, hazard ratio.

^a^ Patients may take multiple types of H1‐antihistamines during the exposure period.

^b^ Patients could be prescribed monotherapy, a combination, or switch of ICIs, as indicated by their ICD‐9‐CM–coded malignancy types.

However, the trend of intermediate use to be independent risk factor for mortality in all patients was inconsistent in patients with liver malignancies (aHR 2.736, 95% CI 1.504–4.978, *p* < 0.001), where it was shown to lead to a statistically significant reduction in mortality risk.

### 3.3 Sensitivity Analysis

See Supporting Information.

## 4 Discussion

We aimed to examine the impact on survival outcomes in patients who are concomitantly treated with ICIs and H1‐antihistamines in the real‐world setting. The interpretation of mortality risk in intermediate users was two‐sided, with a 78% reduction in patients with primary lung malignancies but a 174% increase in patients with primary liver malignancies, both of statistical significance. Frequent use was, however, found to be independently associated with an increased mortality risk of 72% in patients with primary lung malignancies.

The observation of improved survival in patients with lung malignancies receiving ICIs with concomitant H1‐antihistamines is consistent with recent translational research [4], but, unfortunately, a similar survival benefit is not extended to patients with liver malignancies in our study.

One hypothesis is that lung malignancies are typically more aggressive and exhibit a greater propensity for metastasis to various organs compared to liver cancer. Preclinical data [4] indicate that HRH1 blockade enhances the antimetastatic immune response. Additionally, clinical retrospective data [15] support the observation that patients with metastatic cancer who received ICIs in conjunction with antihistamines experienced independently improved survival outcomes.

Although our analyses did not include more specific types of malignancies due to the limited sample size, we are able to make further use of the detailed prescription and dispensing records of H1‐antihistamines to also identify the impact on survival outcomes, considering the generation type and the medication days' supply. Our findings, therefore, fill the research gap, given that these aspects of concomitant medications have remained unexplored to date. The adoption of stringent inclusion criteria and the application of multivariable analysis help minimize bias, and the data from real‐world cohorts represent a spectrum of patients that includes those with multiple comorbidities, thus enabling the results drawn to be more applicable in routine clinical practice.

Major differences among patients' baseline characteristics after baseline are insignificant except for PLR, total bilirubin, and creatinine, and therefore are unlikely to explain the findings. However, patients in the cohort study might be at a rather late or advanced stage of disease progression and have a poor prognosis, evidenced by a median duration of follow‐up of 7.0 months from receiving the first dose of ICIs. This phenomenon is coherent with the usual clinical practice during the period from 2014 to 2018, when most ICIs were approved as second‐line, third‐line, or adjuvant by the United States Food and Drug Administration (FDA) to be integrated into the standard of care across different malignancies [16]. Consequently, ICIs were reserved until treatment failures with multiple prior lines of chemotherapy and/or targeted therapy. The duration of follow‐up might be longer when there are more approved first‐line indications for ICIs.

In this context, the duration of follow‐up is even 2–3 months shorter in intermediate and frequent H1‐antihistamine users compared to minimal and infrequent users. Intermediate and frequent use of H1‐antihistamines might indicate management of allergic conditions due to manifestations of inflammatory responses. The relationship between allergy and malignancy remains unclear and controversial, with some suggesting that chronic allergic inflammation promotes malignancy development and progression, whereas others suggest that enhanced immunosurveillance due to allergy limits malignant transformation [17]. Despite the controversies surrounding this issue, this may provide a potential explanation for why frequent use is independently associated with an increased mortality risk in patients with primary lung malignancies.

Here, the superimposed chronic T cell–mediated allergic inflammation, indicative of redirection of T cells toward a pathogenic and pro‐inflammatory phenotype [18] during ICI treatment, might explain the significantly increased mortality risk despite H1‐antihistamine use in frequent users. The ad hoc prescription of H1‐antihistamines for mild and/or nonspecific allergy symptom management in infrequent users might account for the trend of decreased mortality risk by reversing malignancy‐specific immune evasion and ICI resistance via the histamine–HRH1 axis. The biased nature of agonism of H1‐antihistamines at HRH1, causing receptor desensitization and/or internalization [19] after intermediate and frequent use, might be another plausible explanation for the difference in mortality risk across the user groups.

Both second‐ and third‐generation H1‐antihistamines demonstrate better efficacy and cause fewer neurological side effects than the first generation [5]. The improved receptor selectivity might explain the reduction of mortality risk in second‐ and third‐generation H1‐antihistamine users, despite the statistical insignificance of these findings. Another potential angle would be the effect of antihistamines on the gut microflora [20]. It is possible that these perturbations may lead to significant host–microbiome interactions that affect the efficacy of immunotherapy.

Several caveats of our study should be acknowledged. First, detailed data on the exact causes, whether allergic or nonallergic, for the prescription and dispensing of H1‐antihistamines, as well as the record of any clinically relevant comorbid conditions, including allergic and autoimmune diseases, were not available in our study. These may or may not be documented in the consultation notes. Hence, further analyses on the mechanisms behind the trends of increased mortality risk in frequent users but decreased mortality risk in infrequent users are not feasible. Second, H1‐antihistamines are available both as prescription (Rx) or over‐the‐counter (OTC) medications in Hong Kong [21] and are on the list of USFDA's Rx‐to‐OTC switch [22]. The possibility that patients with allergic disorders might also take OTC H1‐antihistamines could not be eliminated in our study, and information on drug adherence is also absent. However, because hospitalizations are common in patients who are diagnosed with advanced stages of malignancies, their medication adherence can largely be ensured. Third, patients might take multiple generations of H1‐antihistamines during the exposure period, and data on the use of other well‐researched medications, such as antibiotics and aspirin, that alter the effectiveness of ICIs are also not available in our study. Potential synergistic or antagonistic drug–drug interactions might be present. Fourth, the arbitrary selection of inclusion and exclusion criteria might introduce bias. Users here were defined as H1‐antihistamine use 1 month before and after the first dose of ICI, with the assumption that pre‐exposure to H1‐antihistamines before ICI treatment would modify the TME by reverting macrophage immunosuppression and revitalizing T cell function, thus allowing patients to better respond to ICIs. Finally, there are several additional significant confounding factors that may influence treatment response and survival, which were not addressed in our analyses. These factors include, but are not limited to, tumor stage, metastasis, Eastern Cooperative Oncology Group (ECOG) performance status, concurrent or subsequent chemotherapy or radiotherapy, and additional doses of ICIs. As this study is a retrospective cohort analysis, the potential for confounding and bias is likely greater than in prospective studies, where data collection is generally more rigorous and systematic. Moreover, some pertinent information may only be documented in clinical notes, which would not be captured by CDARS, the registry of clinical data for patients receiving public healthcare services in Hong Kong. We acknowledge these limitations and emphasize the need for more comprehensive investigations in future studies on this topic.

In conclusion, our findings are, by and large, in line with previous studies suggesting that H1‐antihistamines could be promising adjuvants to enhance ICI responses in patients with lung malignancies. Unfortunately, H1‐antihistamine use may increase mortality risk in patients with liver malignancies. The increased mortality risk in intermediate or frequent users might suggest that underlying chronic allergic inflammation poses challenges to restoring ICI response. We observed that third‐generation H1‐antihistamines could be independently associated with a reduction in mortality risk among all patients, despite the lack of statistical significance. Further mechanistic studies are necessary to investigate the regimen and generation type of H1‐antihistamines that best enhance ICI response.

## Author Contributions

**Yin Leung:** formal analysis, writing – original draft, writing – review and editing, data curation. **Terry Cheuk‐Fung Yip:** data curation, formal analysis, supervision. **Grace Lai‐Hung Wong:** funding acquisition, supervision, writing – review and editing, validation, conceptualization, project administration. **Vincent Wai‐Sun Wong:** conceptualization, supervision, writing – review and editing, project administration, validation. **Vicki Wing‐Ki Hui:** formal analysis, data curation. **Tony Shu‐Kam Mok:** supervision. **Henry Lik‐Yuen Chan:** supervision. **Stephen Lam Chan:** supervision. **Rashid Nok‐Shun Lui:** conceptualization, writing – review and editing, project administration, supervision.

## Conflicts of Interest

Grace Wong has served as an advisory committee member for AstraZeneca, Gilead Sciences, GlaxoSmithKline, and Janssen, and as a speaker for Abbott, AbbVie, Ascletis, Bristol‐Myers Squibb, Echosens, Gilead Sciences, Janssen, and Roche. She has also received a research grant from Gilead Sciences. Vincent Wong has served as an advisory committee member for 3V‐BIO, AbbVie, Allergan, Echosens, Gilead Sciences, Janssen, Novartis, Novo Nordisk, Perspectum Diagnostics, Pfizer, and Terns; and as a speaker for Bristol‐Myers Squibb, Echosens, Gilead Sciences, and Merck. He has also received a research grant from Gilead Sciences. Terry Yip has served as an advisory committee member and a speaker for Gilead Sciences. Henry Chan has served as a member of the board of directors for Shanghai Henlius Biotech Inc.; as an advisory committee member for AbbVie, Aligos, Aptorum, Arbutus, Hepion, Intellia, Janssen, Gilead, MedImmune, Roche, Vaccitech, VenatoRx, Vir Biotechnology, and GRAIL; and as a speaker for Mylan, Gilead, and Roche. Tony Mok has served as a member of the board of directors for AstraZeneca, Chi‐Med, and Sanomics; has received grants or research support from AstraZeneca, Bristol‐Myers Squibb, Clovis Oncology, Merck Sharp & Dohme, Novartis, Pfizer, Roche, SFJ Pharmaceuticals, and XCovery; speakers' fees from AstraZeneca, Bristol‐Myers Squibb, Boehringer Ingelheim, Eli Lilly, Merck Sharp & Dohme, Novartis, Pfizer, Roche/Genentech, Taiho, and Takeda Oncology; honoraria from ACEA Biosciences, AstraZeneca, Boehringer Ingelheim, Bristol‐Myers Squibb, Celgene, Eli Lilly, Fishawack Facilitate, Ignyta, Janssen, Merck Serono, Merck Sharp & Dohme, Novartis, OncoGenex Pharmaceuticals, Pfizer, Roche/Genentech, SFJ Pharmaceuticals, Takeda Oncology, and Vertex Pharmaceuticals; is a major stockholder in Sanomics; and is an advisory board member for ACEA Biosciences, AstraZeneca, Boehringer Ingelheim, Bristol‐Myers Squibb, Celgene, ChiMed, Cirina, Clovis Oncology, Eli Lilly, Fishawack Facilitate, geneDecode Co., Ignyta, Janssen, Pfizer, Merck Serono, Merck Sharp & Dohme, Novartis, Roche/Genentech, SFJ Pharmaceuticals, Takeda, and Vertex Pharmaceuticals. Stephen Chan has served as an advisory committee member for MSD, AstraZeneca, Eisai, and Ipsen.

## Supporting information


Data S1.

## Data Availability

The data supporting the findings of this study are available in the Supporting Information of this article.
