# Supplementary material for: Concomitant Usage of H1‐Antihistamines and Immune Checkpoint Inhibitors on Cancer Patient Survival
Source: Cancer Med. 2025 Jan 10;14(1):e70583. doi: 10.1002/cam4.70583 (PMC11719706; doi:10.1002/cam4.70583)
Supplement: Supplementary file 1 — Data S1. [file CAM4-14-e70583-s001.docx]

**SUPPLEMENTARY MATERIALS**

Supplementary Table [1]. Malignancy type categorized according to ICD-9-CM (arranged alphabetically)

| **Malignancy type** | **ICD-9-CM** | **Description** |
| --- | --- | --- |
| Breast | 174.3 | Cancer of female breast lower-inner quad |
|  | 174.4 | Cancer of female breast upper-outer |
|  | 174.5 | Cancer of breast, female lower-outer |
|  | 174.6 | Cancer of breast axillary tail |
|  | 174.9 | Cancer of female breast |
|  | 239.3 | Breast tumor |
|  | 150.1 | Cancer of esophagus - thoracic |
| Gastrointestinal | 150.8 | Cancer of esophagus |
|  | 151.0 | Cancer of stomach - cardia |
|  | 151.1 | Cancer of stomach - pylorus |
|  | 151.2 | Cancer of pyloric antrum |
|  | 151.4 | Cancer of stomach - body |
|  | 151.5 | Cancer of stomach lesser curve |
|  | 151.8 | Cancer of stomach |
|  | 151.9 | Cancer of stomach |
|  | 153.1 | Cancer of transverse colon |
|  | 153.2 | Cancer of descending colon |
|  | 153.3 | Cancer of sigmoid colon |
|  | 153.4 | Cancer of cecum |
|  | 153.5 | Cancer of appendix vermiformis |
|  | 153.9 | Cancer of colon |
|  | 154.1 | Cancer of rectum |
|  | 154.3 | Anal cancer |
|  | 157.0 | Cancer of pancreas - head |
|  | 157.1 | Cancer of pancreas - body |
|  | 157.2 | Cancer of pancreas - tail |
|  | 157.8 | Cancer of pancreas |
|  | 157.9 | Cancer of pancreas |
|  | 230.2 | Cancer in situ stomach |
|  | 235.2 | Gastrointestinal stromal tumor, stomach |
| Genital organs | 180.1 | Cancer of exocervix |
|  | 180.9 | Cancer of cervix uteri |
|  | 183.0 | Malignant neoplasm of ovary |
|  | 184.0 | Malignant neoplasm of vagina |
|  | 185 | Cancer of prostate |
|  | 186.9 | Cancer of testis |
|  | 187.7 | Malignant neoplasm of scrotum |
| Hematological | 200.10 | Lymphosarcoma |
|  | 200.12 | Lymphosarcoma, thorax |
|  | 200.18 | Lymphosarcoma, multiple |
|  | 201.50 | Hodgkin's disease, nodular sclerosis |
|  | 201.52 | Hodgkin's disease, nodular sclerosis, thorax |
|  | 201.55 | Hodgkin's disease, nodular sclerosis, inguinal |
|  | 201.58 | Hodgkin's disease, nodular sclerosis, multiple |
|  | 201.60 | Hodgkin's disease, mixed cellularity |
|  | 201.90 | Hodgkin's lymphoma |
|  | 202.00 | Nodular lymphoma |
|  | 202.80 | Malignant lymphoma extranodal/ solid organ sites |
|  | 202.82 | Lymphoma, thorax |
|  | 202.85 | Lymphoma, inguinal |
|  | 202.88 | Lymphoma, multiple sites |
|  | 202.93 | Lymphoid malignancy abdomen |
|  | 203.00 | Multiple myeloma |
|  | 204.00 | Acute lymphoid leukemia |
|  | 205.00 | Acute myeloid leukemia |
|  | 205.01 | Acute myeloid leukemia in complete remission |
|  | 205.30 | Myeloid sarcoma |
|  | 205.90 | Myeloid leukemia |
|  | 208.90 | Leukemia |
| Liver | 155.0 | Cancer of liver, primary |
|  | 155.1 | Cancer of intrahepatic bile duct |
|  | 156.1 | Cancer of extrahepatic bile ducts |
|  | 156.8 | Cancer of gallbladder and extrahepatic bile ducts |
|  | 156.9 | Cancer of biliary tract |
|  | 197.7 | Metastasis to liver |
|  | 239.0 | Liver tumor |
| Lung | 162.2 | Cancer of main bronchus |
|  | 162.3 | Cancer of upper lobe, bronchus/ lung |
|  | 162.4 | Cancer of middle lobe, bronchus/ lung |
|  | 162.5 | Cancer of lower lobe, bronchus/ lung |
|  | 162.8 | Cancer of bronchus or lung |
|  | 162.9 | Cancer of bronchus and lung |
|  | 197.0 | Pulmonary secondary |
|  | 197.2 | Malignant Pleural Effusion |
|  | 235.7 | Neoplasm of uncertain behavior of lung |
| Oral cavity | 141.0 | Cancer of tongue - base |
|  | 141.1 | Cancer of tongue, dorsal surface |
|  | 141.2 | Cancer of tip and lateral border tongue |
|  | 141.9 | Cancer of tongue |
|  | 143.0 | Cancer of upper gum |
|  | 143.1 | Cancer of lower gum |
|  | 144.9 | Cancer of floor of mouth |
|  | 145.6 | Cancer of retromolar area |
|  | 146.0 | Cancer of tonsil |
|  | 146.6 | Cancer of oropharynx, lateral wall |
|  | 146.8 | Cancer of oropharynx |
|  | 146.9 | Cancer of oropharynx |
|  | 147.9 | Cancer of nasopharynx |
|  | 148.1 | Cancer of pyriform sinus |
|  | 148.2 | Cancer of hypopharynx aryepiglottic fold |
|  | 148.8 | Cancer of hypopharynx, other site |
|  | 235.8 | Thymoma |
|  | 239.1 | Vocal cord tumor |
| Others | 158.0 | Cancer of retroperitoneum |
|  | 158.8 | Cancer of peritoneum, pelvic |
|  | 160.0 | Cancer of nasal cavity |
|  | 160.2 | Cancer of maxillary sinus |
|  | 161.0 | Malignant neo glottis |
|  | 164.0 | Cancer of thymus |
|  | 164.2 | Malignant neoplasm of anterior mediastinum |
|  | 164.9 | Cancer of mediastinum |
|  | 170.6 | Cancer of pelvic bone/ sacrum/ coccyx |
|  | 171.0 | Malignant neoplasm of connective and other soft tissue of head, face, and neck |
|  | 171.2 | Malignant neoplasm of connective and other soft tissue of upper limb, including shoulder |
|  | 171.3 | Malignant neoplasm of connective and other soft tissue of lower limb, including hip |
|  | 171.4 | Cancer of connective/ soft tissue thorax |
|  | 171.6 | Cancer of connective/ soft tissue pelvis |
|  | 171.9 | Cancer connective+ soft tissue |
|  | 190.3 | Malignant neoplasm of conjunctiva |
|  | 191.1 | Malignant neoplasm of frontal lobe |
|  | 191.2 | Malignant neoplasm of temporal lobe |
|  | 191.3 | Malignant neoplasm of parietal lobe |
|  | 194.6 | Malignant neoplasm of aortic body and other paraganglia |
|  | 195.1 | Malignant tumor of chest wall |
|  | 196.0 | Metastatic lymph nodes - head & neck |
|  | 196.3 | Metastatic lymph nodes - axilla, upper limb |
|  | 196.9 | Secondary to regional lymph nodes |
|  | 198.3 | Brain metastasis |
|  | 198.5 | Bone metastasis |
|  | 198.7 | Metastasis to adrenal gland |
|  | 198.89 | Metastasis to other specified site |
|  | 199.0 | Metastasis to multiple sites |
|  | 199.1 | Invasive transition cell carcinoma |
|  | 233.4 | Carcinoma in situ of prostate |
|  | 235.1 | Neoplasm of uncertain behavior of lip, oral cavity, and pharynx |
|  | 237.5 | Neoplasm of uncertain behavior of brain and spinal cord |
|  | 237.6 | Neoplasm of uncertain behavior of meninges |
|  | 237.9 | Neoplasm of uncertain behavior of other and unspecified parts of nervous system |
|  | 239.7 | Adrenal tumor |
|  | 239.8 | Pelvic tumor |
|  | 239.9 | Neoplasm of unspecified nature, site unspecified |
| Skin | 172.0 | Malignant melanoma of skin of lip |
|  | 172.4 | Skin melanoma, scalp |
|  | 172.5 | Skin melanoma, chest wall |
|  | 172.6 | Skin melanoma, UL excl. shoulder |
|  | 172.7 | Skin melanoma, foot |
|  | 172.9 | Skin melanoma |
|  | 173.3 | Cancer of skin - face |
|  | 173.5 | Cancer of anus - perianal skin |
|  | 173.6 | Skin cancer of upper limb |
|  | 173.7 | Other and unspecified malignant neoplasm of skin of lower limb, including hip |
|  | 173.9 | Cancer of skin |
| Urinary tract | 188.0 | Cancer of urinary bladder - trigone |
|  | 188.2 | Cancer of urinary bladder lateral wall |
|  | 188.3 | Cancer of urinary bladder anterior wall |
|  | 188.4 | Cancer of urinary bladder poster wall |
|  | 188.5 | Cancer of urinary bladder neck |
|  | 188.6 | Cancer of ureteric orifice |
|  | 188.8 | Cancer of urinary bladder, other site |
|  | 188.9 | Cancer of urinary bladder |
|  | 189.0 | Cancer of kidney, left |
|  | 189.1 | Cancer of kidney - Renal pelvis, left |
|  | 189.2 | Cancer of ureter, right |
|  | 189.8 | Cancer of kidney and ureter |
|  | 198.0 | Metastasis to kidney |
|  | 198.1 | Metastatic cancer bladder |
|  | 239.4 | Bladder neoplasm |
|  | 239.5 | Kidney tumor |

Supplementary Figure [1]. Study flowchart detailing patient identification and inclusion criteria of the 2-month sensitivity analysis
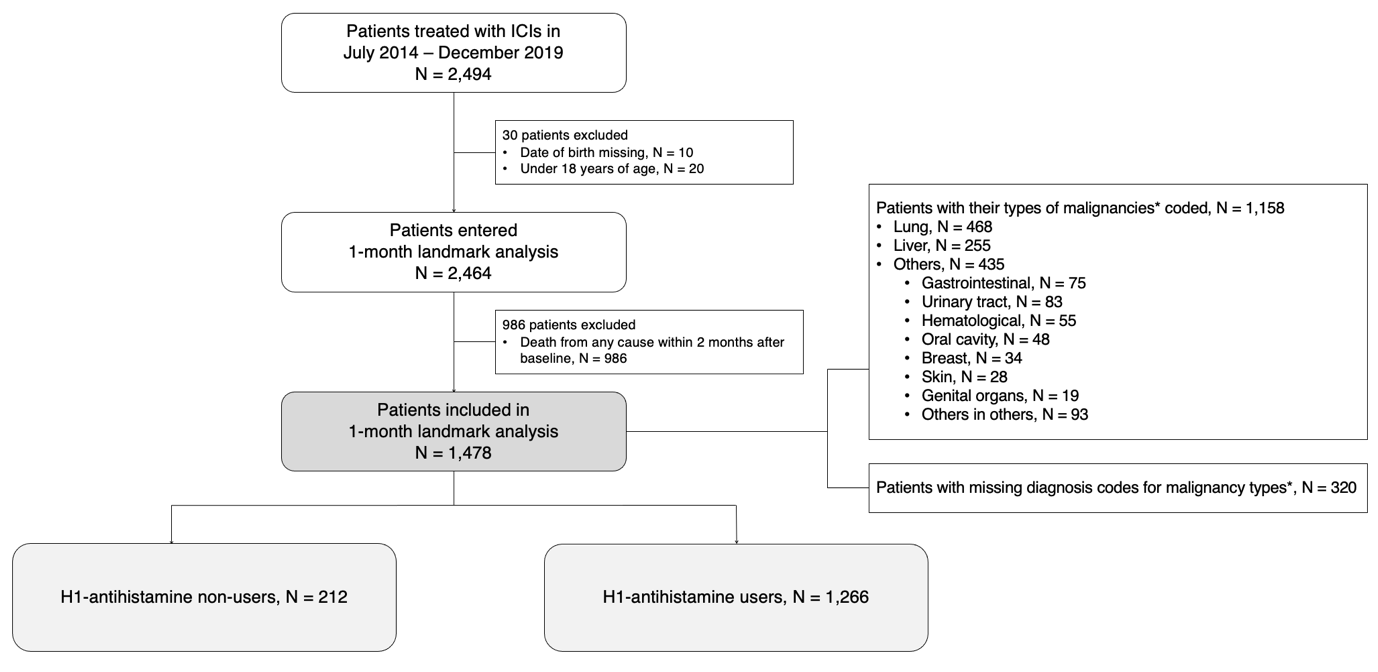


* Categorization of malignancy types was based on the primary site or tissue of the malignant neoplasms only.

Supplementary Table [2]. Baseline characteristics of patients included in the 2-month sensitivity analysis, grouped according to the quartiles of percentages of days’ supply of H1-antihistamines during the exposure period

|  |  | **H1-antihistamine users** | | | |  |
| --- | --- | --- | --- | --- | --- | --- |
|  | **All**  **N=1,478** | **Minimal**  **N=1,179** | **Short-term**  **N=168** | **Medium-term**  **N=69** | **Long-term**  **N=62** | ***P* value** |
| Duration of follow-up (months) ^a^ | 8.8 (4.6-17.4) | 9.1 (4.7-18.2) | 8.4 (5.1-16.4) | 7.1 (4.0-13.4) | 8.3 (4.0-15.1) | 0.191 |
| **Demographic characteristics** |  |  |  |  |  |  |
| Male gender, n (%) | 955 (64.6) | 761 (64.5) | 105 (62.5) | 46 (66.7) | 43 (69.4) | 0.785 |
| Age (years) | 62.1 ± 12.3 | 61.7 ± 12.4 | 62.6 ± 12.8 | 62.7 ± 11.3 | 66.5 ± 10.2 | **0.027** |
| **Clinical characteristics** |  |  |  |  |  |  |
| Hemoglobin (g/dL) | 11.6 ± 1.9 | 11.7 ± 2.0 | 11.6 ± 2.0 | 11.4 ± 1.6 | 11.6 ± 1.5 | 0.754 |
| White cell count (x10^9^/L) | 7.3 ± 4.7 | 7.2 ± 4.4 | 7.4 ± 3.6 | 8.4 ± 9.6 | 7.3 ± 3.6 | 0.250 |
| Neutrophil (x10^9^/L) | 5.1 ± 3.5 | 5.1 ± 3.7 | 5.1 ± 3.0 | 4.8 ± 2.3 | 5.1 ± 3.1 | 0.935 |
| Lymphocyte (x10^9^/L) | 1.3 ± 1.3 | 1.3 ± 1.3 | 1.5 ± 1.5 | 1.2 ± 0.7 | 1.3 ± 0.6 | 0.487 |
| Platelet (x10^9^/L) | 267.2 ± 128.4 | 265.1 ± 126.5 | 273.1 ± 140.5 | 274.0 ± 127.8 | 282.8 ± 132.0 | 0.625 |
| Neutrophil-to-lymphocyte ratio | 4.9 ± 5.2 | 4.9 ± 4.9 | 5.1 ± 6.9 | 6.1 ± 7.2 | 4.3 ± 2.5 | 0.320 |
| Missing (%) |  |  |  |  |  |  |
| Platelet-to-lymphocyte ratio | 256.3 ± 194.4 | 253.6 ± 176.3 | 263.1 ± 293.2 | 305.7 ± 212.9 | 235.1 ± 132.8 | 0.237 |
| Missing (%) |  |  |  |  |  |  |
| International normalized ratio | 1.1 ± 0.2 | 1.1 ± 0.2 | 1.1 ± 0.1 | 1.1 ± 0.1 | 1.1 ± 0.2 | 0.800 |
| C-reactive protein (mg/dL) | 45.2 ± 53.0 | 42.3 ± 50.1 | 58.6 ± 56.9 | 71.2 ± 77.2 | 27.0 ± 36.7 | **0.017** |
| Albumin (g/L) | 36.5 ± 6.1 | 36.6 ± 6.0 | 35.7 ± 6.4 | 35.5 ± 7.0 | 36.1 ± 5.9 | 0.134 |
| C-reactive-protein-to-albumin ratio | 1.5 ± 2.0 | 1.3 ± 1.8 | 1.9 ± 1.9 | 2.5 ± 3.9 | 0.8 ± 1.0 | **0.018** |
| Total bilirubin (μmol/L) | 11.2 ± 22.1 | 11.2 ± 22.3 | 12.0 ± 22.7 | 13.4 ± 25.2 | 8.3 ± 6.7 | 0.587 |
| Alanine aminotransferase (U/L) | 21.0 (14.0-35.0) | 21.0 (14.0-35.0) | 21.0 (13.0-36.0) | 21.0 (15.0-44.0) | 21.5 (16.0-33.0) | 0.401 |
| Aspartate aminotransferase (U/L) | 36.0 (24.0-85.0) | 37.0 (24.0-84.0) | 45.0 (25.5-120.0) | 37.0 (27.0-48.0) | 28.1 (24.0-38.2) | 0.053 |
| Missing (%) | 66.0 | 66.2 | 64.9 | 75.4 | 54.8 |  |
| Creatinine (μmol/L) | 79.1 ± 39.7 | 78.3 ± 34.4 | 77.3 ± 28.7 | 83.0 ± 36.5 | 95.8 ± 105.9 | **0.006** |
| Alpha-fetoprotein (μg/L) | 3.1 (2.0-17.6) | 3.2 (2.0-25.7) | 3.1 (2.0-10.9) | 2.5 (1.8-4.7) | 2.5 (2.1-4.4) | 0.367 |
| Missing (%) | 53.5 | 53.4 | 56.5 | 44.9 | 56.5 |  |
| Positive HBsAg, n (%) ^b^ | 58 (16.1) | 43 (15.6) | 9 (18.4) | 3 (23.1) | 3 (13.0) | 0.836 |
| Missing (%) | 75.6 | 76.7 | 70.8 | 81.2 | 62.9 |  |
| Positive anti-HCV, n (%) ^b^ | 4 (1.7) | 3 (1.6) | 1 (4.0) | 0 (0.0) | 0 (0.0) | 0.749 |
| Missing (%) | 84.2 | 84.4 | 85.1 | 87.0 | 74.2 |  |
| **ICI type, n (%) ^c^** |  |  |  |  |  |  |
| PD-1 |  |  |  |  |  |  |
| Pembrolizumab | 779 (52.7) | 609 (51.7) | 97 (57.7) | 43 (62.3) | 30 (48.4) | 0.179 |
| Nivolumab | 500 (33.8) | 407 (34.5) | 52 (31.0) | 19 (27.5) | 22 (35.5) | 0.463 |
| PD-L1 |  |  |  |  |  |  |
| Atezolizumab | 218 (14.7) | 180 (15.3) | 21 (12.5) | 5 (7.2) | 12 (19.4) | 0.158 |
| Avelumab | 2 (0.1) | 1 (0.1) | 1 (0.6) | 0 (0.0) | 0 (0.0) | 0.397 |
| Durvalumab | 6 (0.4) | 5 (0.4) | 1 (0.6) | 0 (0.0) | 0 (0.0) | 0.876 |
| CTLA-4 |  |  |  |  |  |  |
| Ipilimumab | 140 (9.5) | 108 (9.2) | 21 (12.5) | 5 (7.2) | 6 (9.7) | 0.542 |
| **ICI regimen, n (%)** |  |  |  |  |  |  |
| PD-1 alone | 1045 (70.7) | 826 (70.1) | 119 (70.8) | 56 (81.2) | 44 (71.0) | 0.261 |
| PD-L1 alone | 185 (12.5) | 152 (12.9) | 19 (11.3) | 5 (7.2) | 9 (14.5) | 0.482 |
| CTLA-4 alone | 1 (0.1) | 1 (0.1) | 0 (0.0) | 0 (0.0) | 0 (0.0 | 0.968 |
| PD-1/PD-L1 ± CTLA-4 | 1230 (83.2) | 978 (83.0) | 138 (82.1) | 61 (88.4) | 53 (85.5) | 0.512 |
| *Note*: Duration of follow-up, alanine aminotransferase, aspartate aminotransferase and alpha-fetoprotein were expressed in median (interquartile range), whereas other continuous variables were expressed in mean ± standard deviation. *P*-values < .05 were underlined and made bold.  *Abbreviations*: HBsAg, hepatitis B surface antigen; anti-HCV, antibody to hepatitis C virus; ICI, immune checkpoint inhibitor.  ^a^ Duration of follow-up was the duration from the date of first prescription of ICI to the date of death from any cause, censored at the date of their last prescription of H1-antihistamines before 1 June 2022.  ^b^ Percentages were based on non-missing data only.  ^c^ Patients may have combined or switching ICIs during the follow-up period. | | | | | | |

Supplementary Table [3]. Types of H1-antihistamine use in the cohort of the 2-month sensitivity analysis

|  |  | **Type of malignancy of patients ^a^** | | |
| --- | --- | --- | --- | --- |
|  | **All (N=1,478)** | **Lung (N=468)** | **Liver (N=255)** | **Missing or others (N=755)** |
| **First-generation** | **1,238 (83.8)** | **385 (82.3)** | **220 (86.3)** | **633 (83.8)** |
| Chlorpheniramine ^b^ | 1,211 (81.9) | 379 (81.0) | 213 (83.5) | 619 (82.0) |
| Hydroxyzine | 219 (14.8) | 52 (11.1) | 41 (16.1) | 126 (16.7) |
| Promethazine | 191 (12.9) | 72 (15.4) | 31 (12.2) | 88 (11.7) |
| Diphenhydramine | 143 (9.7) | 48 (10.3) | 12 (4.7) | 83 (11.0) |
| **Second-generation** | **411 (27.8)** | **136 (29.1)** | **61 (24.0)** | **214 (28.3)** |
| Loratadine | 342 (23.1) | 113 (24.1) | 49 (19.2) | 180 (23.8) |
| Cetirizine | 137 (9.3) | 44 (9.4) | 22 (8.6) | 71 (9.4) |
| **Third-generation** | **86 (5.8)** | **18 (3.8)** | **21 (8.2)** | **47 (6.2)** |
| Fexofenadine | 86 (5.8) | 18 (3.8) | 21 (8.2) | 47 (6.2) |
| **Generation switch ^c^** | **430 (29.1)** | **135 (28.8)** | **71 (27.8)** | **224 (29.7)** |
| ^a^ Categorization of malignancy types was based on the primary site or tissue of the malignant neoplasms only.  ^b^ Chlorpheniramine included chlorpheniramine and dexchlorpheniramine.  ^c^ Patients may switch to a different generation of H1-antihistamines for more than once during the exposure period. | | | | |

Supplementary Table [4]. Univariate and multivariate analyses on factors associated with death from any cause in 6 months after receiving the first dose of ICI with an exposure period to H1-antihistamines 2 months before and after the baseline

|  | **Univariate analysis** | | **Multivariate analysis** | |
| --- | --- | --- | --- | --- |
|  | **HR (95% CI)** | ***P*** | **aHR (95% CI)** | ***P*** |
| **All patients (N=1,478)** |  |  |  |  |
| Age (years) | 0.990 (0.988-1.001) | 0.117 | 0.993 (0.985-1.000) | **0.043** |
| Male gender, n | 0.970 (0.814-1.165) | 0.769 | 0.984 (0.816-1.188) | 0.868 |
| Neutrophil-to-lymphocyte ratio | 1.030 (1.020-1.043) | **<.001** |  |  |
| Platelet-to-lymphocyte ratio | 1.000 (1.000-1.001) | **0.005** |  |  |
| By H1-antihistamine days’ supply |  |  |  |  |
| Minimal user | Referent |  |  |  |
| Short-term user | 1.021 (0.777-1.342) | 0.879 | 1.078 (0.809-1.435) | 0.608 |
| Medium-term user | 1.482 (1.034-2.125) | **0.032** | 1.500 (1.026-2.192) | **0.036** |
| Long-term user | 1.133 (0.751-1.711) | 0.551 | 1.232 (0.801-1.896) | 0.343 |
| By H1-anthistamine generation type |  |  |  |  |
| First-generation user | 1.040 (0.822-1.317) | 0.742 | 1.040 (0.807-1.342) | 0.761 |
| Second-generation user | 1.000 (0.826-1.211) | 0.996 | 0.983 (0.801-1.207) | 0.871 |
| Third-generation user | 0.690 (0.453-1.045) | 0.079 | 0.652 (0.417-1.019) | 0.060 |
| By ICI type ^b^ |  |  |  |  |
| PD-1 | 0.860 (0.672-1.111) | 0.254 | 0.413 (0.192-0.890) | **0.024** |
| PD-L1 | 0.990 (0.777-1.262) | 0.936 | 0.450 (0.215-0.941) | **0.034** |
| CTLA-4 | 0.620 (0.437-0.885) | **0.008** | 0.640 (0.447-0.917) | **0.015** |
| **Patients with lung malignancies (N=468)** | |  |  |  |
| Age (years) | 1.000 (0.981-1.013) | 0.699 | 1.001 (0.981-1.021) | 0.928 |
| Male gender, n | 0.790 (0.541-1.139) | 0.203 | 0.663 (0.423-1.037) | 0.072 |
| Neutrophil-to-lymphocyte ratio | 1.020 (0.992-1.040) | 0.193 | 1.016 (0.987-1.045) | 0.296 |
| Platelet-to-lymphocyte ratio | 1.000 (1.000-1.001) | 0.272 |  |  |
| By H1-antihistamine days’ supply |  |  |  |  |
| Minimal user | Referent |  |  |  |
| Short-term user | 0.628 (0.327-1.205) | 0.162 | 0.902 (0.446-1.823) | 0.773 |
| Medium-term user | 0.771 (0.314-1.896) | 0.572 | 0.908 (0.276-2.981) | 0.873 |
| Long-term user | 1.315 (0.664-2.606) | 0.432 | 1.818 (0.795-4.159) | 0.157 |
| By H1-anthistamine generation type |  |  |  |  |
| First-generation user | 0.980 (0.613-1.573) | 0.939 | 1.014 (0.569-1.806) | 0.962 |
| Second-generation user | 0.830 (0.548-1.243) | 0.359 | 0.688 (0.411-1.152) | 0.155 |
| Third-generation user | 0.610 (0.193-1.910) | 0.393 | 0.590 (0.138-2.520) | 0.477 |
| By ICI type ^b^ |  |  |  |  |
| PD-1 | 0.590 (0.397-0.862) | **0.007** | 0.396 (0.092-1.711) | 0.215 |
| PD-L1 | 1.560 (1.066-2.283) | **0.022** | 0.757 (0.177-3.231) | 0.707 |
| **Patients with liver malignancies (N=255)** | |  |  |  |
| Age (years) | 1.000 (0.986-1.017) | 0.873 | 0.993 (0.977-1.010) | 0.423 |
| Male gender, n | 0.900 (0.559-1.465) | 0.684 | 0.967 (0.579-1.617) | 0.899 |
| Neutrophil-to-lymphocyte ratio | 1.070 (0.990-1.159) | 0.088 |  |  |
| Platelet-to-lymphocyte ratio | 1.000 (1.001-1.003) | **0.002** |  |  |
| By H1-antihistamine days’ supply |  |  |  |  |
| Minimal user | Referent |  |  |  |
| Short-term user | 1.623 (0.917-2.869) | 0.096 | 1.796 (0.958-3.367) | 0.068 |
| Medium-term user | 3.237 (1.490-7.031) | **0.003** | 3.362 (1.487-7.604) | **0.004** |
| Long-term user | 0.776 (0.191-3.159) | 0.723 | 0.886 (0.211-3.724) | 0.869 |
| By H1-anthistamine generation type |  |  |  |  |
| First-generation user | 1.160 (0.636-2.127) | 0.623 | 0.965 (0.473-1.971) | 0.923 |
| Second-generation user | 1.300 (0.840-2.015) | 0.239 | 1.228 (0.772-1.954) | 0.386 |
| Third-generation user | 0.770 (0.357-1.659) | 0.504 | 0.601 (0.264-1.368) | 0.225 |
| By ICI type ^b^ |  |  |  |  |
| PD-1 | 1.550 (0.217-11.151) | 0.661 | 0.191 (0.015-2.455) | 0.204 |
| PD-L1 | 0.170 (0.024-1.213) | 0.077 | 0.086 (0.007-1.090) | 0.058 |
| CTLA-4 | 0.760 (0.466-1.233) | 0.264 | 0.690 (0.420-1.134) | 0.143 |
| **Patients with missing diagnosis codes or other malignancies (N=755)** | | | | |
| Age (years) | 0.990 (0.986-1.003) | 0.227 | 0.993 (0.984-1.002) | 0.133 |
| Male gender, n | 1.120 (0.889-1.408) | 0.339 | 1.111 (0.871-1.416) | 0.398 |
| Neutrophil-to-lymphocyte ratio | 1.050 (1.039-1.067) | **<.001** |  |  |
| Platelet-to-lymphocyte ratio | 1.000 (1.000-1.002) | **0.003** |  |  |
| By H1-antihistamine days’ supply |  |  |  |  |
| Minimal user | Referent |  |  |  |
| Short-term user | 1.089 (0.763-1.553) | 0.640 | 1.182 (0.808-1.728) | 0.390 |
| Medium-term user | 1.620 (1.026-2.557) | **0.039** | 1.598 (0.979-2.607) | 0.061 |
| Long-term user | 1.218 (0.697-2.129) | 0.490 | 1.273 (0.706-2.299) | 0.422 |
| By H1-anthistamine generation type |  |  |  |  |
| First-generation user | 1.010 (0.744-1.373) | 0.946 | 1.014 (0.730-1.407) | 0.935 |
| Second-generation user | 1.020 (0.793-1.306) | 0.888 | 1.000 (0.765-1.304) | 0.994 |
| Third-generation user | 0.600 (0.346-1.053) | 0.075 | 0.564 (0.305-1.044) | 0.068 |
| By ICI type ^b^ |  |  |  |  |
| PD-1 | 0.820 (0.566-1.194) | 0.303 | 0.539 (0.190-1.532) | 0.246 |
| PD-L1 | 1.070 (0.750-1.523) | 0.712 | 0.609 (0.226-1.639) | 0.326 |
| CTLA-4 | 0.390 (0.218-0.693) | **0.001** | 0.417 (0.232-0.747) | **0.003** |
| HR, hazard ratio; aHR, adjusted hazard ratio; CI, confidence interval.  *P*-values < .05 were underlined and made bold.  ^a^ Patients may take multiple types of H1-antihistamines during the exposure period.  ^b^ Patients could be prescribed with monotherapy, combination or switching of ICIs indicated by their ICD-9-CM-coded malignancy types. | | | | |

Supplementary Figure [2]. Kaplan-Meier survival curves of 2-month sensitivity analysis in (a) all patients, (b) patients with lung malignancies, (c) patients with liver malignancies, (d) patients with missing diagnosis codes or other malignancies according to the quartiles of days’ supply of H1-antihistamines

| (a)  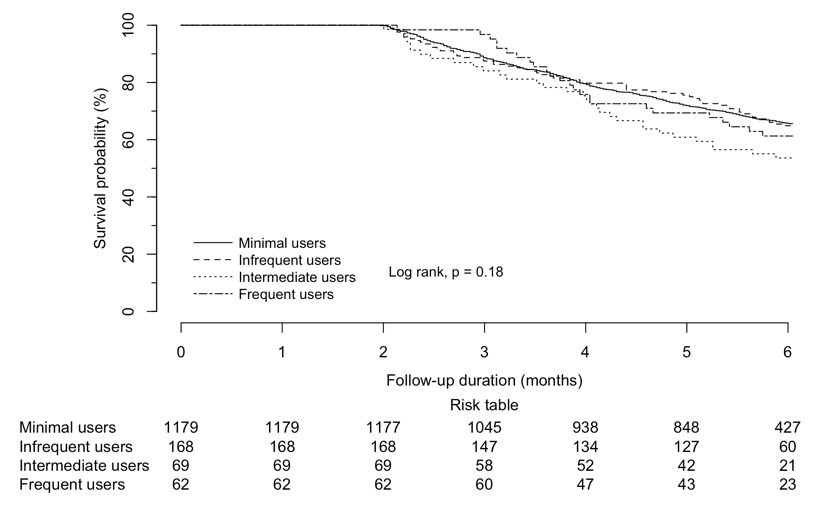 | (b)  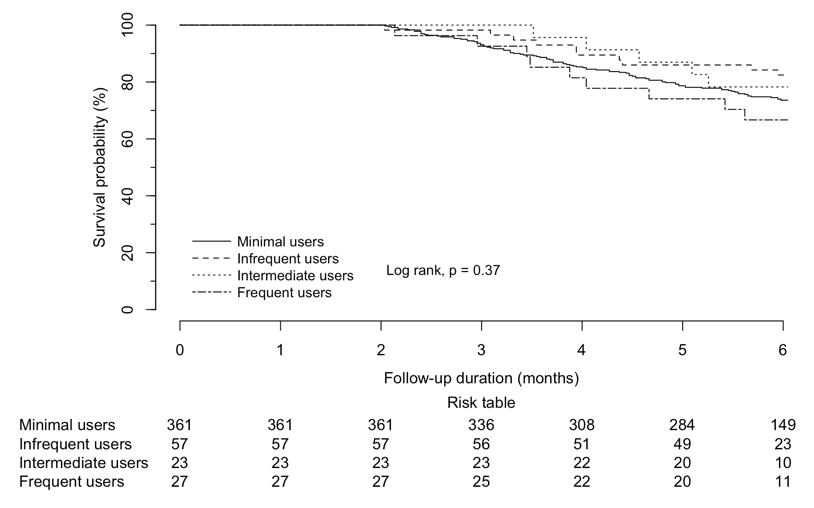 |
| --- | --- |
| (c)  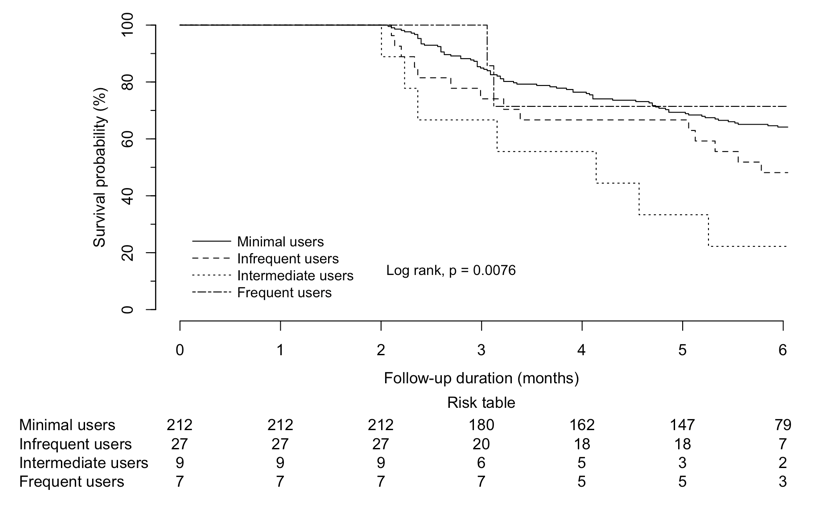 | (d)  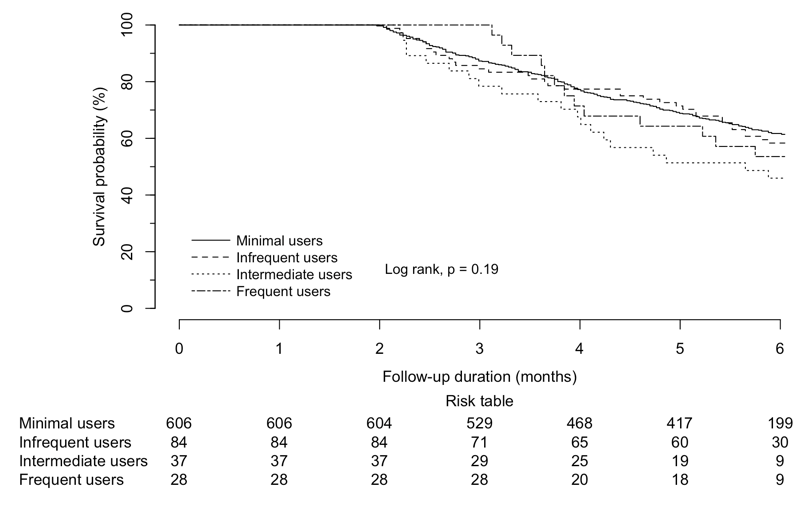 |
